# Supplementary material for: Structure of inhibitor-bound mammalian complex I
Source: Nat Commun. 2020 Oct 16;11:5261. doi: 10.1038/s41467-020-18950-3 (PMC7567858; doi:10.1038/s41467-020-18950-3)
Supplement: Supplementary file 1 — Supplementary Information [file 41467_2020_18950_MOESM1_ESM.pdf]

Supplementary Information

Supplementary Table 1. Cryo-EM data collection, refinement and validation statistics.

	**Piericidin-K2**<br>EMD-11424<br>PDB 6ZTQ	**Piericidin-FIII**<br>EMD-11425	**Active**<br>EMD-11377<br>PDB 6ZR2
**Data collection and processing**			
Magnification	47,600	130,000	47,600
Voltage (kV)	300	300	300
Electron exposure (e–/Å^2)	50	46	50
Defocus range (µm)	-2.2 to -3.4	-2.2 to -3.8	-1.5 to -3.1
Pixel size (Å)	1.050	1.063	1.054
Symmetry imposed	None	None	None
Initial particle images (no.)	60,107	76,802	60,851
Final particle images (no.)	27,193	36,759	20,370
Map resolution (Å)	3.0	3.0	3.1
FSC threshold	0.143	0.143	0.143
Map resolution range (Å)	2.8 to 472	2.8 to 478	2.8 to 474
**Refinement**			
Initial model used	PDB 6G2J		PDB 6G2J
Model resolution (Å)	3.1		3.2
FSC threshold	0.5		0.5
Map sharpening *B* factor (Å^2)	-28	-68	-29
Model composition			
Nonhydrogen atoms	67,069		67,069
Protein residues	8,181		8,181
Ligands	36		35
B factors mean (Å^2)			
Protein	33.6		37.21
Ligand	31.1		35.14
R.m.s. deviations			
Bond lengths (Å)	0.008		0.005
Bond angles (°)	0.869		0.801
Validation			
MolProbity score	1.77		1.70
Clashscore	5.33		4.95
Poor rotamers (%)	0.07		0.07
Ramachandran plot			
Favored (%)	92.06		93.08
Allowed (%)	7.90		6.87
Disallowed (%)	0.04		0.05

Supplementary Table 2. Summary of the models for the subunits of mouse complex I.

Subunit	Alternative names	Chain	Total residues	Modeled residues	% Modeled	Notes
**NDUFV1**	51 kDa, Nqo1, NuoF	F	444	9-436	96.4	FMN, 4Fe4S
**NDUFV2**	24 kDa, Nqo2, NuoE	E	217	5-216	97.7	2Fe2S
**NDUFS1**	75 kDa, Nqo3, NuoG	G	704	6-693	97.7	2Fe2S, 2 x 4Fe4S
**NDUFS2**	49 kDa, Nqo4, NuoCD	D	430	1-430	100	Dimethyl-Arg85
**NDUFS3**	30 kDa, Nqo5, NuoCD	C	228	7-213	90.8	
**NDUFS7**	PSST, Nqo6, NuoB	B	189	34-189	82.5	4Fe4S
**NDUFS8**	TYKY, Nqo9, NuoI	I	178	1-178	100	2 x 4Fe4S
**ND1**	Nqo8, NuoH	H	318	1-318	100	N-formyl
**ND2**	Nqo14, NuoN	N	345	1-344	99.7	N-formyl
**ND3**	Nqo7, NuoA	A	115	1-115	100	N-formyl
**ND4**	Nqo13, NuoM	M	459	1-459	100	N-formyl
**ND4L**	Nqo11, NuoK	K	98	1-98	100	N-formyl
**ND5**	Nqo12, NuoL	L	607	1-606	99.8	N-formyl
**ND6**	Nqo10, NuoJ	J	172	1-171	99.4	N-formyl
**NDUFV3**	10 kDa	s	69	28-68	59.4	
**NDUFS4**	18 kDa	Q	133	9-133	94.0	
**NDUFS5**	15 kDa	e	105	1-105	100	2 x Cys-Cys
**NDUFS6**	13 kDa	R	96	1-94	97.9	Zn^{2+}
**NDUFA1**	MWFE	a	70	1-68	97.1	
**NDUFA2**	B8	S	98	13-95	84.7	
**NDUFA3**	B9	b	83	4-83	96.3	
**NDUFA5**	B13	V	115	2-115	99.1	
**NDUFA6**	B14	W	130	17-130	87.7	
**NDUFA7**	B14.5a	r	112	1-77, 90-112	89.2	N-acetyl
**NDUFA8**	PGIV	X	171	1-171	100	4 x Cys-Cys
**NDUFA9**	39 kDa	P	342	1-342	100	NADPH
**NDUFA10**	42 kDa	O	320	1-320	100	ATP
**NDUFA11**	B14.7	Y	142	3-142	98.6	Cys-Cys (2x in active)
**NDUFA12**	B17.2	q	145	1-144	99.3	N-acetyl
**NDUFA13**	B16.6	Z	143	3-143	98.6	
**NDUFAB1α**	SDAPα	T	88	7-82	86.3	4'-phosphopantethine + 3-hydroxyundecanoate
**NDUFAB1β**	SDAPβ	U	88	3-88	97.7	
**NDUFB1**	MNLL	f	56	4-56	94.6	
**NDUFB2**	AGGG	j	72	7-68	86.1	
**NDUFB3**	B12	k	103	19-93	72.8	
**NDUFB4**	B15	m	128	3-128	98.4	
**NDUFB5**	SGDH	h	143	6-143	96.5	
**NDUFB6**	B17	i	127	1-36, 66-123	74.0	N-acetyl
**NDUFB7**	B18	o	136	2-112	81.6	Cys-Cys
**NDUFB8**	ASHI	l	157	3-156	98.1	
**NDUFB9**	B22	n	178	1-177	99.4	
**NDUFB10**	PDSW	p	175	4-172	96.6	2 x Cys-Cys
**NDUFB11**	ESSS	g	122	21-121	82.7	
**NDUFC1**	KFYI	c	49	1-48	98.0	
**NDUFC2**	B14.5b	d	120	1-120	100	

Supplementary Table 3. Parameters from the mechanistic models used to test the kinetic data.

Model		K_M (μM)<br>$(k_{-1}+k_2)/k_1$	k_{cat} (s^{-1})<br>k_2	$K_{I,1}$ (μM)<br>k_{-3}/k_3	$K_{I,2}$<br>k_{-5}/k_5	Additional parameter	SSR
2-site competitive [a]	*Mean*<br>*Median*<br>*95% CI*	979<br>978<br>889 – 1081	338<br>338<br>330 – 346	538<br>500<br>379 – 1074	37.1 nM<br>36.6 nM<br>19 – 49 nM		46.67
2-site uncompetitive [b]	*Mean*<br>*Median*<br>*95% CI*	1122<br>1122<br>1007 – 1238	347<br>347<br>339 – 357	296<br>169<br>48 – 1123	1401 nM<br>954 nM<br>139 – 4937 nM		49.21
Mixed	*Mean*<br>*Median*<br>*95% CI*	1058<br>1057<br>873 – 1267	364<br>364<br>351 – 380	88.3<br>33.5<br>10 – 973	15.9 μM<br>15.7 μM<br>13 – 20 μM		121.87
Uncompetitive [c]	*Mean*<br>*Median*<br>*95% CI*	1118<br>1116<br>943 – 1301	369<br>368<br>355 – 384	14.1<br>14.1<br>12 – 16			128.33
Product-state competitive	*Fit*<br>*95% CI*	472 mM<br>334 – 610 mM	372<br>353 – 391	13.09<br>11 – 15		k_{-6}/k_6 =<br>108.24<br>0 – 906	128.44
Competitive [d]	*Mean*<br>*Median*<br>*95% CI*	902<br>892<br>712 – 1116	351<br>351<br>334 – 371	1.61<br>1.59<br>1.23 – 2.02			200.98
Competitive (+ in-facing) [e]	*Fit*<br>*95% CI*	932<br>675 – 1095	354<br>332 – 369	1.67<br>1.01 – 2.19		K_{in} =<br>155 mM<br>0 – 835 M	201.08

Models deemed worthwhile were bootstrapped (see Methods) to obtain mean, median and 95% confidence intervals for parameter values. Those not bootstrapped are reported as fitting values with a 95% confidence interval derived from the fitting algorithm.

a – $K_{I,1}$ and $K_{I,2}$ were allowed to take any value. Scheme shown in Fig. 5.

b – The inhibitor can bind twice (as for 2-site competitive) but only to the enzyme-substrate complex.

c – The inhibitor can bind only to the enzyme-substrate complex.

d – Scheme shown in Fig. 5.

e – The inhibitor was also allowed to bind to enzyme that is catalytically inactive because its NADH binding site faces the proteoliposome lumen.

Supplementary Table 4. List of molecular simulations. Sites are defined by the centre-of-mass distances between the piericidin and Tyr108 rings: site **1** 2-5 Å; site **1'** 10-15 Å; site **2** 20-25 Å, and site **2'** 25-30 Å. MD, classical atomistic molecular dynamics simulations (total 240 ns); QM/MM, hybrid quantum/classical molecular dynamics simulations (total 1.5 ps); CG, coarse-grained molecular dynamics simulations (total 100 μs).

Simulation type	Molecule	Site occupancy	Simulation
MD	2 P	1+2	A1 (60 ns), A2 (40 ns)
MD	P	1	A3 (40 ns)
MD	2 P	1+2	A4 (40 ns)
MD	P	2	A5 (40 ns)
MD	2P, His59-NδH$^+$	1+2	A6 (20 ns)
QM/MM	P	1	B1 (1.5 ps)
CG	P	1	C1 (10 μs)
CG	P	1	C2 (10 μs)
CG	P	1'	C3 (10 μs)
CG	P	1'	C4 (10 μs)
CG	P	2	C5 (10 μs)
CG	P	2	C6 (10 μs)
CG	P	2'	C7 (10 μs)
CG	P	2'	C8 (10 μs)
CG	P	2'/out	C9 (10 μs)
CG	P	2'/out	C10 (10 μs)

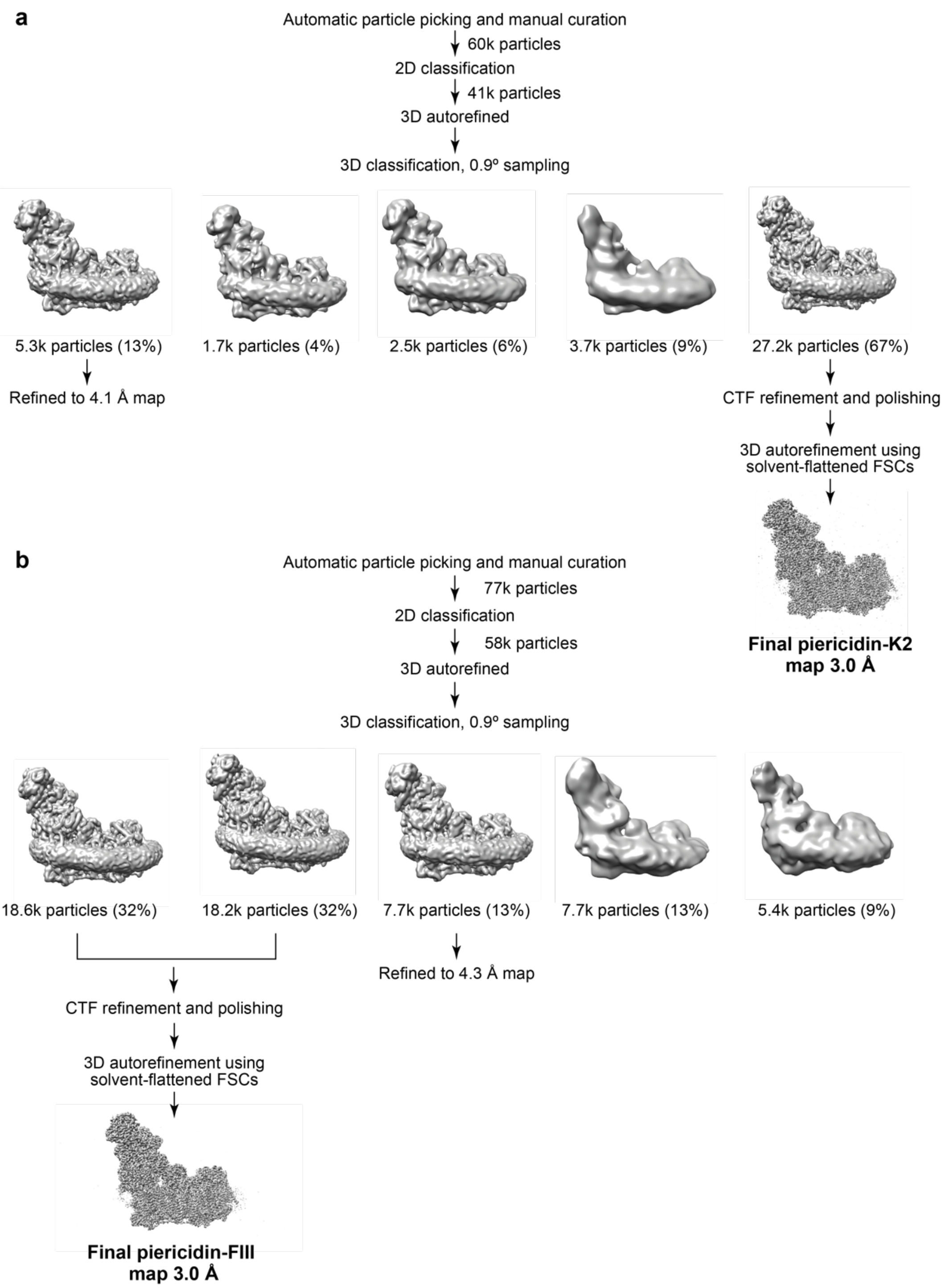

Supplementary Fig. 1 | Classification and refinement of the mouse complex I piericidin-K2 and piericidin-FIII cryo-EM density maps.

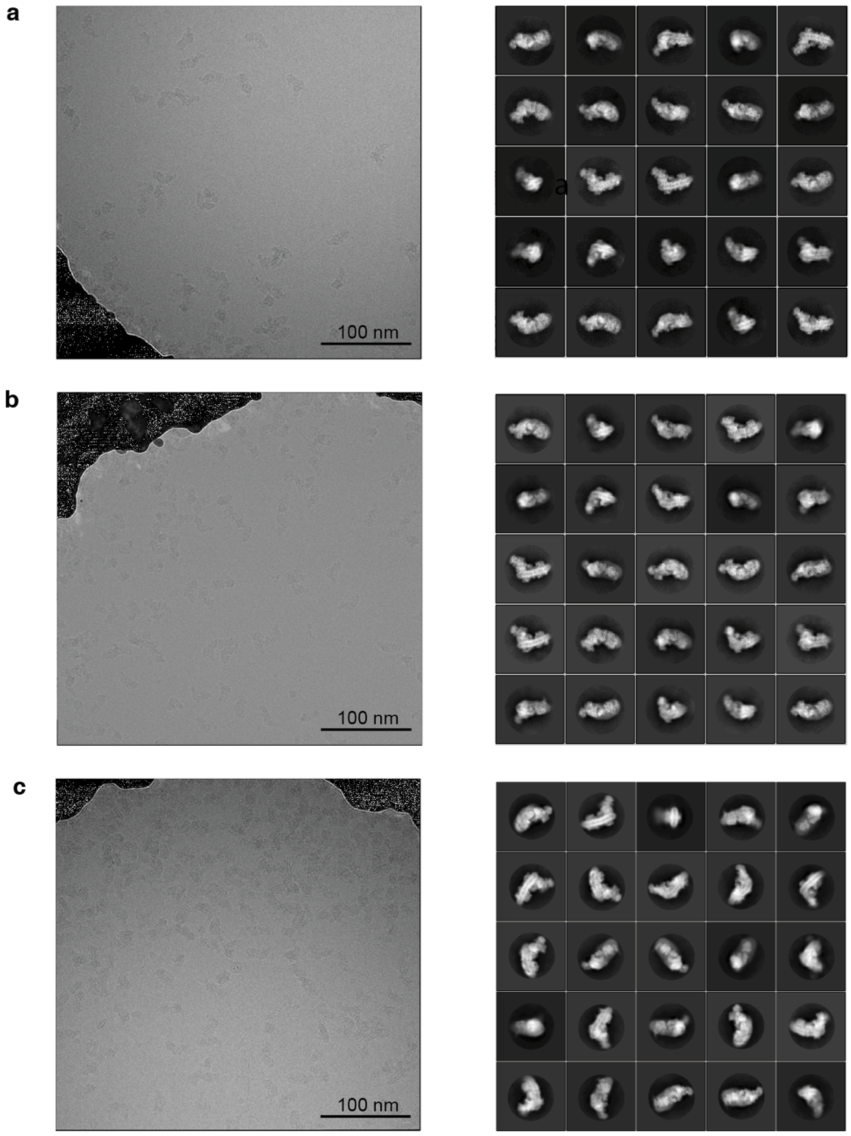

Supplementary Fig. 2 | Example micrographs and 2D classes from the cryo-EM datasets. a) piericidin-K2, b) piericidin-F3 and c) active enzyme. The micrograph images have been Motion and CTF corrected and low-pass filtered to 20 Å resolution. A total of 1200, 1454 and 1235 micrographs were recorded, respectively. Example views were selected following 2D classification of the final 3D refined particles to show classes of particles in different orientations.

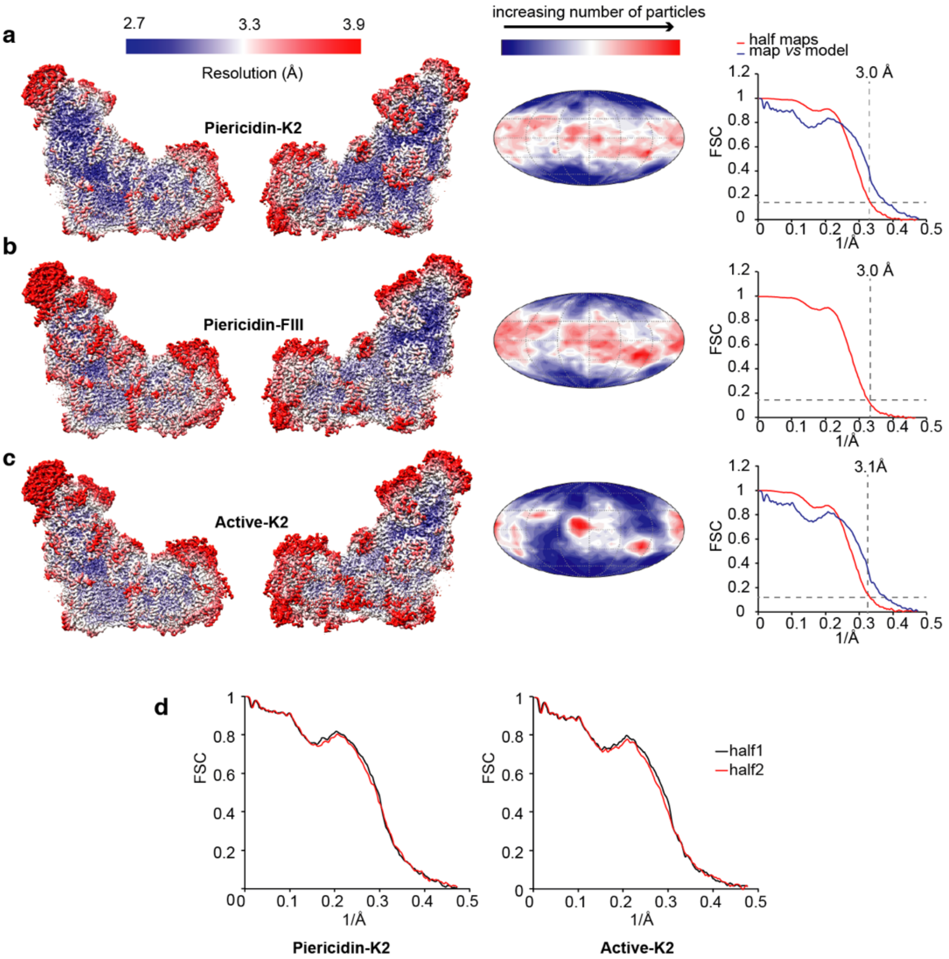

Supplementary Fig. 3 | Resolution estimates of the maps for the active and piericidin-bound states of complex I, particle orientation distributions, and cross-validation of Phenix real space refinement parameters. Data are for the **a)** piericidin-K2 dataset, **b)** piericidin-FIII dataset **c)** active enzyme. The estimated resolutions, defined where the red FSC curve = 0.143, are 3.0, 3.0 and 3.1 Å, respectively. For calculation of the orientation distributions, each dimension was split into 30 bins and the angles of rotation and tilt are taken from the '_rlnAngleRot' and '_rlnAngleTilt' values for each particle after refinement in RELION. The models agree well with their respective maps, as shown by the map *vs*. model FSC curves (blue). Local resolutions were estimated using the Local Resolution function in RELION with default parameters and plotted with UCSF Chimera using contour levels of 0.032, 0.062 and 0.032 in a, b and c respectively. **d)** One of the two half maps was used for refinement, then FSC curves were calculated for each half map using the same model.

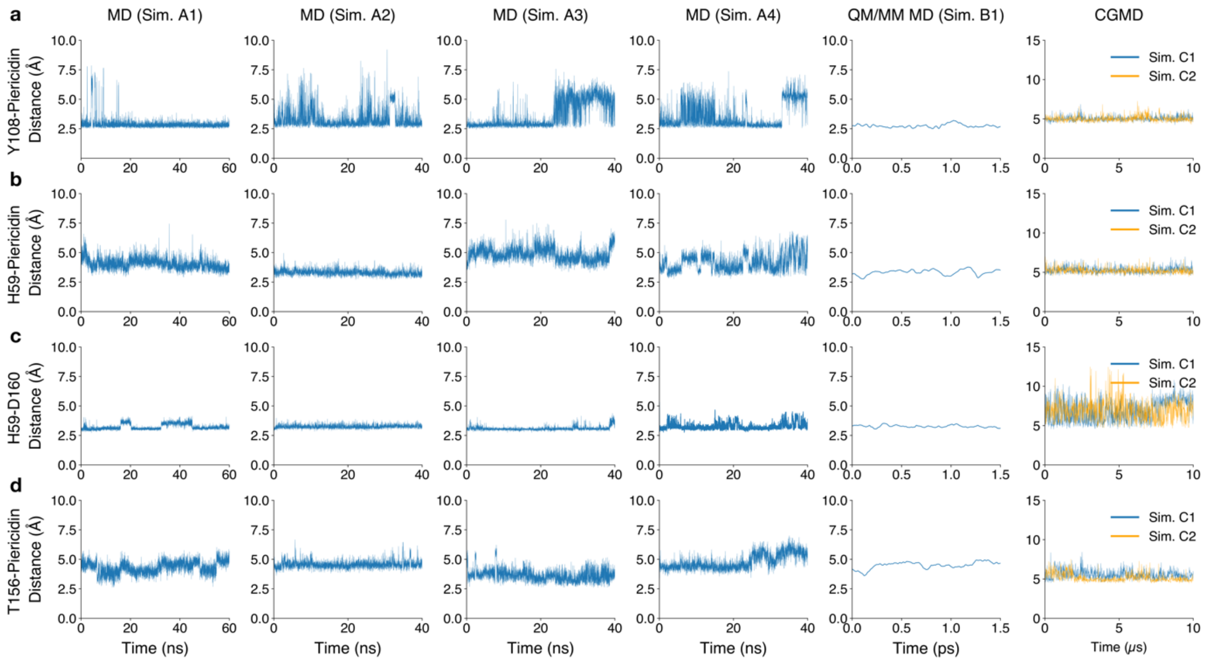

Supplementary Fig. 4 | Distances between piericidin in site 1 and surrounding residues obtained from classical MD, QM/MM MD, and coarse-grained MD simulations. See Fig. 3a main text for structural information. Classical MD simulations are shown in columns 1-4 (simulation A1-A4, Supplementary Table 4), QM/MM MD in column 5 (simulation B1), and coarse-grained (CGMD) simulations in column 6 (simulations C1-C10). **a)** Piericidin 4' carbonyl oxygen to Tyr108 hydroxyl hydrogen. **b)** Piericidin 2' methoxy oxygen to His59 (centre-of-mass of Nδ/Cε/Nε). **c)** His59 (centre-of-mass of Nδ/Cε/Nε) to Asp160 (centre-of-mass of carboxylic group). **d)** Piericidin 2' methoxy oxygen to Thr156 hydroxyl oxygen.

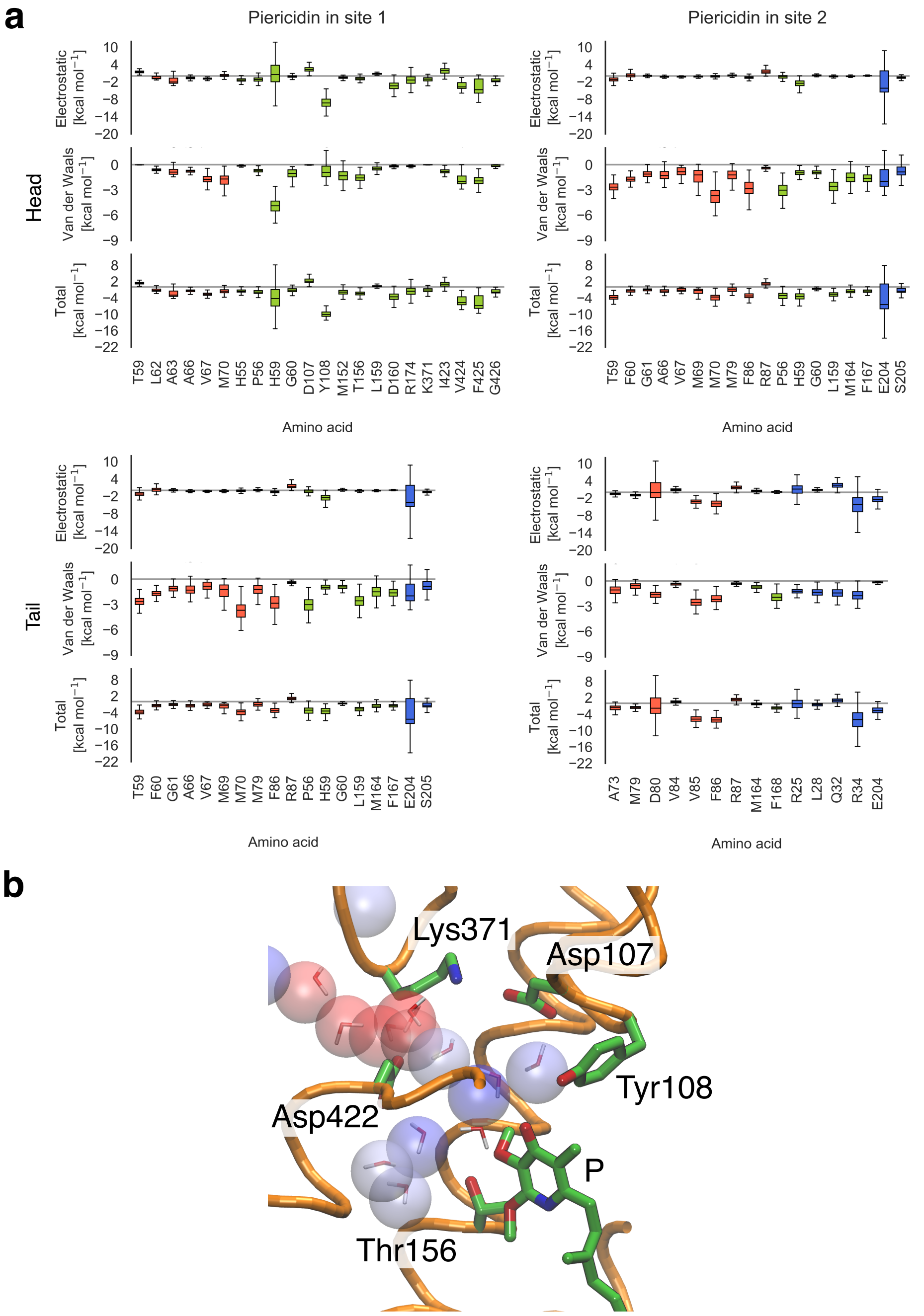

Supplementary Fig. 5. | Interaction energies between the bound piericidin molecules and surrounding protein residues and water clustering analysis during classical MD simulations. a) The non-bonded interaction energies are from simulations A3 (piericidin site 1) and A1 (piericidin site 2). Red, NDUFS7; green, NDUFS2; blue, ND1. The boxes extend from the 25th to the 75th percentile, the middle line represents the median. The whiskers show the range of the data from the 10th to 90th percentile (n = 1500, snapshots calculated every 40 ps). The head group (top row) and tail (lower row) are separated between the 6’ – 1 carbons. Note that the reported values represent interactions energies, as estimation of binding free energies are outside the scope of the present work. **b)** Water molecules predicted to be present in the cavity between Lys371, Asp422, Asp107, and Thr156 using the WATCLUST algorithm (last 40 ns of simulation A1, see Methods). The clusters (spheres) are coloured according to the water occupancy from 0 (red) to 1 (blue). The structure and water molecules shown are a representative snapshot of water occupation during MD simulation A1 (see Supplementary Table 4).
