# Supplementary material for: Structure of inhibitor-bound mammalian complex I
Source: Nat Commun. 2020 Oct 16;11:5261. doi: 10.1038/s41467-020-18950-3 (PMC7567858; doi:10.1038/s41467-020-18950-3)
Supplement: Supplementary file 3 — Reporting Summary [file 41467_2020_18950_MOESM3_ESM.pdf]

nature research

Corresponding author(s): Ville Kaila, Judy Hirst

Last updated by author(s): Sep 10, 2020

Reporting Summary

Nature Research wishes to improve the reproducibility of the work that we publish. This form provides structure for consistency and transparency in reporting. For further information on Nature Research policies, see Authors & Referees and the Editorial Policy Checklist .

Statistics

For all statistical analyses, confirm that the following items are present in the figure legend, table legend, main text, or Methods section.

n/a	Confirmed	
[ ]	[x]	The exact sample size (*n*) for each experimental group/condition, given as a discrete number and unit of measurement
[ ]	[x]	A statement on whether measurements were taken from distinct samples or whether the same sample was measured repeatedly
[x]	[ ]	The statistical test(s) used AND whether they are one- or two-sided<br>*Only common tests should be described solely by name; describe more complex techniques in the Methods section.*
[x]	[ ]	A description of all covariates tested
[x]	[ ]	A description of any assumptions or corrections, such as tests of normality and adjustment for multiple comparisons
[ ]	[x]	A full description of the statistical parameters including central tendency (e.g. means) or other basic estimates (e.g. regression coefficient) AND variation (e.g. standard deviation) or associated estimates of uncertainty (e.g. confidence intervals)
[x]	[ ]	For null hypothesis testing, the test statistic (e.g. *F*, *t*, *r*) with confidence intervals, effect sizes, degrees of freedom and *P* value noted<br>*Give P values as exact values whenever suitable.*
[x]	[ ]	For Bayesian analysis, information on the choice of priors and Markov chain Monte Carlo settings
[x]	[ ]	For hierarchical and complex designs, identification of the appropriate level for tests and full reporting of outcomes
[x]	[ ]	Estimates of effect sizes (e.g. Cohen's *d*, Pearson's *r*), indicating how they were calculated

Our web collection on statistics for biologists contains articles on many of the points above.

Software and code

Policy information about availability of computer code

Data collection	EPU 1.9, Softmax Pro 5.4.42.1, Bruker Xepr
Data analysis	RELION-2.1-patchb1 and v3.0, MotionCor2, GCTF-1.06, Phenix 1.13-2998 and 1.16-3549 , Coot-0.8.9.1/0.9-pre, EMRinger, MolProbity 4.4, SCIPION 1.2, AceDRG, UCSF Chimera-1.13.1, PyMol-1.8.4.0 and 2.2.3, MATLAB 2018a, NAMD-2.9/2.13, VMD, WATCLUST, Gromacs 2016.3, CHARMM c38b , TURBOMOLE v.6.6-7.3, CHARMM/TURBOMOLE python interface

For manuscripts utilizing custom algorithms or software that are central to the research but not yet described in published literature, software must be made available to editors/reviewers. We strongly encourage code deposition in a community repository (e.g. GitHub). See the Nature Research guidelines for submitting code & software for further information.

Data

Policy information about availability of data

All manuscripts must include a data availability statement. This statement should provide the following information, where applicable:

- Accession codes, unique identifiers, or web links for publicly available datasets
- A list of figures that have associated raw data
- A description of any restrictions on data availability

The source data underlying Figs. 3, 4, 5 and Supplementary Figs. 3, 4, 5 are provided as a Source Data file. Data accession codes: EMD-11424, PDB ID: 6ZTQ [https://doi.org/10.2210/pdb6ZTQ/pdb] (piericidin-bound structure), EMD-11425 (piericidin-bound map from the FIII detector), EMD-11377, PDB ID: 6ZR2 [https://doi.org/10.2210/pdb6ZR2/pdb] (active state structure). Other data supporting the findings of this manuscript are available from the corresponding authors upon reasonable request.

Field-specific reporting

Please select the one below that is the best fit for your research. If you are not sure, read the appropriate sections before making your selection.

[x] Life sciences   [ ] Behavioural & social sciences   [ ] Ecological, evolutionary & environmental sciences

For a reference copy of the document with all sections, see nature.com/documents/nr-reporting-summary-flat.pdf

Life sciences study design

All studies must disclose on these points even when the disclosure is negative.

Sample size	cryoEM: CryoEM data were recorded in two datasets originating from a single protein purification. Entire data sets: 60,107 (K2) and 76,802 (Falcon III) particles collected and filtered by 2D and 3D classification. Final classes contained 27,193 (K2) and 36,759 (Falcon III) particles. EPR: Single samples of each condition were prepared due to high sample requirements. Kinetics and analytical methods: three to six technical replicates of each data point were sufficient to provide the accuracy required.
Data exclusions	CryoEM: 2D images were picked automatically and classified, then data for non-protein or damaged protein images were removed (see Methods). No data were excluded from biochemical or computational experiments.
Replication	cryoEM data were recorded in two data sets. MD simulations were repeated in duplicates. Kinetic measurements and analytical methods were of 3-6 technical replicates. EPR was measured on a single sample per condition. No replicate experiments have been excluded.
Randomization	Not relevant to the experimental design
Blinding	Not relevant to the experimental design

Reporting for specific materials, systems and methods

We require information from authors about some types of materials, experimental systems and methods used in many studies. Here, indicate whether each material, system or method listed is relevant to your study. If you are not sure if a list item applies to your research, read the appropriate section before selecting a response.

Materials & experimental systems

n/a	Involved in the study	
[x]	[ ]	Antibodies
[x]	[ ]	Eukaryotic cell lines
[x]	[ ]	Palaeontology
[ ]	[x]	Animals and other organisms
[x]	[ ]	Human research participants
[x]	[ ]	Clinical data

Methods

n/a	Involved in the study	
[x]	[ ]	ChIP-seq
[x]	[ ]	Flow cytometry
[x]	[ ]	MRI-based neuroimaging

Animals and other organisms

Policy information about studies involving animals; ARRIVE guidelines recommended for reporting animal research

Laboratory animals	Six-week old female C57BL/6 mice
Wild animals	N/A
Field-collected samples	N/A
Ethics oversight	Mice were sacrificed by cervical dislocation in accordance with the UK Animals (Scientific Procedures) Act, 1986 (PPL: 70/7538, approved by the local ethics committees of the MRC Laboratory of Molecular Biology and the University of Cambridge and by the UK Home Office) and the University of Cambridge Aminal Welfare Policy. The work involved only schedule 1 procedures and wild-type animals.

Note that full information on the approval of the study protocol must also be provided in the manuscript.
